# Supplementary material for: Are carbon emissions trading and green financial instruments synergistic? -Comprehensive quantitative research based on content analysis
Source: PLoS One. 2024 Mar 7;19(3):e0298601. doi: 10.1371/journal.pone.0298601 (PMC10919601; doi:10.1371/journal.pone.0298601)
Supplement: S1 Data — (ZIP) [file pone.0298601.s001.zip › Data/地级市Z_F工作报告.zip!/╝¬┴╓/│ñ┤║╩╨/└┤╫╘╣1⁄2╓┌║┼í╛┬φ┐╦╩2╛▌═°í┐_2004.docx]

各位代表：

　　 我代表市人民政府向大会作工作报告，请予审议。并请列席会议的同志提出意见。

　　 一、2003年工作的简要回顾

　　 过去的一年，我们在省委、省政府和市委的正确领导下，在市人大、市政协的监督和支持下，坚持以邓小平理论和“三个代表”重要思想为指导，全面贯彻十六大精神，依靠全市各族人民，推进改革开放和现代化建设，保持了经济社会全面发展的良好势头，各项工作取得了新成绩。

　　 （一）全力做好防治工作，抗击非典斗争取得阶段性重要成果。去年上半年，面对突如其来的非典疫情灾害，我们紧紧依靠市委的坚强领导，把人民群众的身体健康和生命安全放在第一位，把防治工作作为各项工作的重中之重，迅速建立起有力、有序、有效的指挥领导系统。实行全民动员，依法防治，群防群控，构建了科学严密的治、控、防综合体系。市人大常委会在关键时刻紧急审议通过了《关于预防和控制传染性非典型肺炎的决定》，为依法防治提供了有力的法律保障。整合各级各类医疗救治资源，紧急组建非典诊疗中心，近千名医护人员舍生忘死，全力以赴投入医疗救治第一线；数千名卫生工作者在公安干警的配合下，依法流调3300多人，随访2万多人（次），医学观察2900多人，有效控制了疫情蔓延和扩散。在城区，各级机关干部带头昼夜坚守在机场、火车站、公路出入口，认真进行卫生安全检查；5万多名干部群众逐家逐户开展“地毯式”排查，形成了横向到边、纵向到底的排查网络和卫生安全监控体系。在农村，15万名干部群众齐上阵，盯防到户，包保到人，构建了严密的防控网络，有效截断了疫情向农村输入。全市物资供应部门克服各种困难，积极组织防治短缺物资的生产和供应，提供了有力的后勤保障。各级宣传部门和新闻媒体，坚持正确的舆论导向，及时、客观地公布疫情和防治动态，加强防治知识宣传，提供了有力的思想舆论支持。各级领导干部和广大共产党员在关键时刻，率先垂范，深入防治第一线，坚定了人民群众战胜疫情灾害的信心。驻长人民解放军、武警部队和一汽、吉大、电力、长铁分局、民航管理局、南航北方吉林分公司以及省直医疗、防疫等部门，自觉服从属地化管理，形成了抗击非典的强大合力。社会各界、各兄弟城市和海内外朋友慷慨解囊，无私援助，捐款捐物折合人民币4000多万元。在做好防治工作的同时，我们认真总结抗非经验教训，切实加强公共卫生管理、监督和投入，提高了群众卫生防护意识；建立健全各种预警和应急机制，落实经常性防治措施，增强了应对各种突发事件和风险的能力。在这场斗争中，全市各族人民众志成城，和衷共济，对政府在非常时期采取的非常措施给予充分理解和支持，体现了空前的凝聚力和战斗力，共同铸就了“临危不惧、沉着应对，实事求是、尊重科学，无私奉献、顽强拼搏，万众一心、敢于胜利”的抗非精神，已经成为推动长春全面振兴的强大精神动力！

　　 （二）坚持加快发展不动摇，国民经济持续快速健康发展。全市实现国内生产总值1338亿元，比上年增长14.2%。实现全口径财政收入134.8亿元，增长22.3%。完成全社会固定资产投资389.6亿元，增长21.6%。农业生产战胜了严重春旱，粮食取得好收成，总产量达670万吨。粮食销售突破500万吨，增长1倍多。大力发展生态农业和畜牧业，启动农产品质量标准体系和检验检测体系建设，新建无公害和绿色食品种植基地25个、标准化牧业小区163个。畜牧业实现增加值81.2亿元，占一产增加值的52.1%。农业产业化进程加快，东高大豆、新希望乳制品等20个投资3000万元以上的农产品加工项目建成投产。县域经济发展明显加快。四县（市）和双阳区实现国内生产总值405.8亿元、全口径财政收入11.5亿元,分别增长16.5%和8.4%。积极实施“治水兴屯”工程，新打机电井143眼，治理水土流失22万亩，农业生产条件得到改善。城镇建设稳步推进。新增城镇道路468.9万平方米、燃气管道 24.5公里。新修乡村公路1304公里，297个村屯通油（水泥）路。75万农民外出务工经商,农民增收渠道进一步拓宽。工业经济速度和效益同步增长。工业实现增加值542.3亿元，增长17.1%。规模以上工业实现利润突破100亿元，增长24.1%。四大主导行业进一步优化升级。奥迪A4、马自达M6、丰田陆地巡洋舰等具有国际先进水平的产品相继投放市场，汽车工业实现产值1181亿元，占全市规模以上工业产值的78.2%。光电子信息、生物与医药产业快速发展。高新技术产业实现产值520亿元，占规模以上工业产值比重超过1/3。重点项目建设进展良好，新建续建投资超亿元工业重点项目22个，一汽“三大基地”（一汽轿车新基地、一汽--大众轿车二厂、解放卡车新基地）、大成德惠玉米生化工业园等具有百亿元产值前景的特大项目开工建设，进一步增强了经济发展后劲。建筑业实现增加值88亿元，增长18%。流通业现代化步伐加快，连锁经营已经扩大到餐饮、图书、医药等多个业种。实现全社会消费品零售总额438.3亿元，增长8.4%。旅游业实现总收入83亿元，增长23.8%，占全省旅游业总收入的58%。会展经济成果喜人，国际雕塑大会、净月冰雪旅游节吸引了世界的目光，汽博会、农博会、民博会得到国内外广泛认可，参会参展人数（次）均超过100万。

　　 （三）各项改革稳步推进，对外开放取得新成绩。国企改革取得新进展。20户国有企业完成产权制度改革，16户企业国有资本全部退出。所有制结构进一步优化。民营经济完成固定资产投资84亿元，增长10.5%；实现增加值446亿元，增长20%，占全市国内生产总值的33.3%，提高1.2个百分点。有效利用资本市场，欧亚、经开、亚泰3家上市公司实现配股，融资9.18亿元。积极稳妥推进乡镇事业单位机构改革，削减乡镇站所105个，减员2787人。住房、人事、分配制度等各项改革进一步深化，机关、高校后勤体制改革初见成效，要素市场不断完善。对外开放成效突出，对外贸易快速增长。精心策划、周密安排各项招商活动，成功举办了首次网上招商会，先后组团赴日本、瑞典、俄罗斯、香港等多个国家和地区招商引资。住友等5家世界500强企业和中国海外集团等一批国内知名企业来长落户。全市实际利用外资7.5亿美元、内资145亿元人民币，分别增长19.8%和16.5%。实现外贸进出口总额52.2亿美元，增长80.7%，其中出口15.5亿美元，增长21.9%。努力扩大对外交往，与朝鲜清津市缔结为友好城市。开发区对全市经济的带动作用进一步增强。高新、经开等5个开发区实现国内生产总值498亿元，占全市国内生产总值的37.2%，提高3.2个百分点。九台东湖等6个开发区被批准为省级开发区，四县（市）和双阳区增加了新的发展平台。

　　 （四）城市建设步伐加快，承载能力明显增强。全年投入36亿元，实施70多项城市重点工程。西三环路、黑嘴桥等“八路五桥”按计划完成年度任务，建成民康路等9条标准街路，龙家堡机场、“三北”工程、“轻铁二期”等跨年度工程进展顺利。着眼于城市的可持续发展，启动一批生态建设重点工程。波罗湖湿地生态恢复、西部和西北部防风御沙林工程顺利推进。全市退耕还林7000公顷。世界雕塑公园按期对外开放，南湖公园完成年度改造任务。城区植树29.1万株，新增绿地121公顷，人均公共绿地达7.3平方米。城市环境空气质量优良天数达335天，优级天数增加21天。城市管理综合执法体制顺利运行。拆除“三小”、清理马路市场等综合整治活动深入开展，城区管理职能作用进一步发挥，城市整体形象得到提升。土地市场进一步规范，实现土地净收益8亿元，增长58.8%。超前谋划城市生产力发展布局，调整城区、开发区管理体制，科学界定各自功能，有效拓展城市发展空间，为经济社会持续健康发展奠定了良好基础。

　　 （五）精神文明建设不断深入，各项社会事业全面发展。广泛开展群众性精神文明创建活动，文明社区、文明单位、文明村镇、军（警）民共建等创建活动稳步推进。创建全国文明城市工作通过中央文明办的中试测评。贯彻落实《公民道德建设实施纲要》，深入开展“革除十大陋习、树立文明新风”主题宣传教育活动，市民的文明素质进一步提高。军政军民关系不断密切，创建“双拥模范城”喜获“五连冠”。把社会事业摆到与经济发展同等重要位置，增加了对社会事业的投入。公共卫生设施建设得到加强。新建市传染病医院，启动了北方肝胆医院建设。改建扩建了四县（市）和双阳区疾控中心。教育事业发展较快。素质教育迈出新步伐，全市中小学全部实施新课程改革。新建中小学校舍33.8万平方米、农村教育实践培训基地20个，改造倒危校舍6.85万平方米，城乡办学条件进一步改善。优质高中建设取得新进展，城区高中在校生达44270人，扩招26.6%。职业教育保持良好发展势头，扩招10%，毕业生就业率达96%。高等教育健康发展，人才培养和科技转化能力进一步增强。大力实施“人才兴业”战略，建设学习型城市取得新成效。实施各类培训9.8万人（次），45万人参与市民外语工程，引进各类急需和紧缺人才1.2万人。亚冬会各项筹备工作全面展开，组委会正式成立并履职运行。体育基础设施建设进度加快。莲花山滑雪场等比赛场馆建设进展良好，市滑冰馆改扩建工程竣工，市民健身中心、2座体育健身园、17条健身路径投入使用。竞技体育取得新成绩，长春亚泰队喜获全国足球甲B联赛冠军。启动了长影世纪城、伪满皇宫二期复原等一批重点文化项目，长春图书馆改扩建后达到国家一类一级标准，数字电视顺利试播。坚持不懈地开展爱国卫生运动，居民楼道卫生整治和通行安全管理取得明显成效，创建“国家卫生城”工作通过国家爱卫会可行性调研检查。社区建设成效突出。新建社区服务用房271处，总面积15万平方米，并实现文体、卫生、法律、就业、社保等服务功能入社区。人口与计划生育工作取得新进展，人口自然增长率控制在3.31‰，10县（市）区的计生服务站得到改造。为少数民族办实事，建成第二回族公墓及殡葬服务站。新闻出版、妇女儿童、人防、地震等事业健康发展。援藏工作取得新进展，首批对口援建项目建成并投入使用。

　　 （六）人民生活水平稳步提高，就业和社会保障体系建设取得新进展。城市居民人均可支配收入7905元，农民人均纯收入3411元，分别增长13.5%和8.4%。城乡居民储蓄存款835亿元，比年初增加120亿元，人均增加1678元。居民消费结构不断升级。城镇和农村居民恩格尔系数分别下降0.4和0.6个百分点，教育、文化、娱乐支出的比重提高1.8个百分点，每万户拥有电脑增加253台，每万人拥有移动电话增加140部。汽车和住房成为消费新亮点。新增私人轿 车10693辆，增长23.7%。销售商品住宅185万平方米，市区、农村居民住房人均建筑面积21.94和18.95平方米，分别增加0.74和0.45平方米。全年新增就业岗位8.8万个，5.4万名下岗失业人员和1.35万名“4050”人员实现再就业。17.3万城市居民得到最低生活保障救助。养老、医疗、失业保险覆盖面进一步扩大，参保人数分别新增5.5万、17万和5.1万人；养老失业保险基金征缴突破20亿元，增加3亿多元。企业退休人员社会化管理率达96%。认真解决水、路、房、医等群众日常生活中的热点、难点问题。全年改造二次供水设施305处。亮化67条居民区背街暗巷，改造90条巷路。采取多种措施，安置历史超期回迁居民1437户。600户特困居民住房得到解决，2527名特困患者得到医疗救助，25000多名城乡贫困学生得到资助和减免学费，1950名残疾人通过“扶残助业工程”实现就业。在积极争取国家和省支持的同时，市直26个部门积极对营城煤矿沉陷区实行对口帮扶，解决水、电、路等方面近百个问题，各种投入折合人民币4500多万元。

　　 （七）经济发展软环境不断优化，民主法制建设进一步加强。围绕政府转变职能，提速提效，狠抓经济发展软环境建设。公安、卫生等13个部门“学习经济技术开发区海关”试点工作取得良好成效。政务中心服务功能进一步完善，行政审批效率明显提高。政务中心205项审批核准事项实现“一站式”办理，全年共办理各类审批88502件，当天办结率达86%。把政务公开、纠风专项治理和减轻农民负担作为改善软环境的重点任务，查处273起涉软案件，依法依规处分230人，为企业减负9000多万元，农民人均减负45元。深入整顿和规范市场经济秩序，信用体系建设取得新进展，被列为“全国中小企业信用体系建设试点城市”。城市投资环境不断改善。在世界银行2003年对中国23个有代表性大中城市投资环境的综合评估中，长春居第7位。

　　 自觉接受人大及其常委会的工作监督、法律监督和政协的民主监督，认真接受各级人大代表和政协委员视察、检查57次，办理人大议案、建议、意见155件，政协建议案、提案335件。提请人大审议地方性法规7件，制定行政规章7件。把实行政府采购作为推进廉政建设的重要手段，组织公开招投标702次，节约财政资金4000多万元。努力扩大社会各界的知情权和参与度，政府决策的科学化、民主化和透明度进一步提高。认真倾听群众呼声，“12345”市长公开电话办理群众来电106968件，办结率达96.2%。认真落实信访责任制，加强人民内部矛盾的排查调处工作，一批热点、难点信访问题得到解决。高度重视社会稳定问题，加强消防、交通、安全生产和食品、药品专项整治，严厉打击“法轮功”等邪教组织，深入开展社会治安综合治理，刑事案件发案率下降20.9%。政法战线广大干警奋力拼搏，克难攻坚，为保护人民群众生命财产安全和维护社会稳定做出了突出贡献。

　　 上述成绩的取得，是市委正确领导、统揽全局的结果，是市人大、市政协有效监督和支持的结果，是全市人民团结奋斗、努力拼搏的结果。在此，我谨代表市人民政府，向辛勤工作在各条战线的广大干部群众，向给予政府工作积极支持的人大代表和政协委员，向各民主党派、工商联和各社会团体，向中、省直驻长单位，向中国人民解放军和武警驻长部队官兵，向所有支持长春建设和发展，特别是在防治非典关键时刻给予我们大力支持的海内外朋友，表示诚挚的感谢！

　　 在充分肯定成绩的同时，我们也清醒地看到，我市经济社会发展和政府工作还存在不少困难和问题。市场化程度低，经济发展活力不足；国企改革任务仍很艰巨，城乡二元结构还没有根本改变；非国有经济比重偏低，民营经济规模过小；固定资产投资率低于全国平均水平，对经济拉动作用不足；公共卫生设施建设薄弱，社会公共安全体系亟待健全；就业和社会保障压力增大，部分群众生活还比较困难，农民收入水平仍然偏低；群众关心的一些热点、难点问题还没有得到解决；政府管理体制和运行机制还不适应市场经济发展需要，经济发展软环境尚需进一步改善；政府机关中形式主义、官僚主义和服务意识不强、行政效率不高等问题依然存在；廉政建设尚需进一步加强；安全生产方面的问题也不可忽视，等等。对这些问题我们将高度重视，并采取更加有力的措施，认真加以解决。

　　 二、把握机遇，开拓创新，全力推进长春老工业基地的改造振兴

　　 中央作出振兴东北等老工业基地的战略决策，给长春发展带来了千载难逢的历史机遇。把握和利用好这个机遇，是历史和时代赋予本届政府的神圣使命。必须树立比以往任何时候都更加强烈的忧患意识和发展意识，解放思想，更新观念，增强责任感和紧迫感，以高昂的斗志、积极的态度、务实的作风和科学的精神，全力推进长春老工业基地的改造振兴。

　　 长春老工业基地振兴的总体思路是：以邓小平理论、“三个代表”重要思想和十六大精神为指导，全面贯彻落实十六届三中全会、省委八届四次全会和市委十届四次全会精神，坚持以全面建设小康社会为总目标，坚持建设“三大中心”、发展“四大主导行业”、实施“五大战略”的总体方向，以体制创新、机制创新、科技创新为动力，以经济结构调整为主线，深化国有企业改革，扩大对内对外开放，大力发展民营经济，努力壮大县域经济，优化经济发展环境，力争用7年左右时间，把长春建设成为技术先进、结构合理、功能完善、特色鲜明、竞争力强的新型产业基地。坚持“五个统筹”原则，全面推进社会主义物质文明、政治文明、精神文明协调发展，努力走出一条全面、协调、可持续的振兴之路。

　　 长春老工业基地振兴的具体目标是：到2005年，调整改造初见成效，老工业基地突出矛盾得到缓解。到2007年，调整改造取得显著成果。到2010年，基本完成老工业基地调整改造任务。全市国内生产总值达到3000亿元以上，人均国内生产总值达到38000元左右。全社会固定资产投资累计完成4400亿元左右。城市居民人均可支配收入15000元左右，农民人均纯收入6000元左右。城镇登记失业率控制在5%以内。把长春建设成为国内最具规模的汽车及零部件生产加工出口基地，国内一流的绿色食品产业基地，全国著名的光电子信息产业基地，全国重要的生物医药和中药现代化产业基地，面向东北乃至东北亚地区的区域性物流产业基地，成为吉林省乃至东北地区全面振兴的重要增长极。

　　 2004年，是全面实施振兴老工业基地战略的起步年，也是十分关键的一年。必须扎扎实实地做好各项工作，确保开好局、起好步，为实现长春老工业基地振兴目标打下坚实基础。今年全市国内生产总值预期目标是1520亿元，增长13%左右。

　　 （一）积极推进国企改革，增强长春老工业基地振兴的内在动力。以建立现代产权制度为核心，加快国企改革，着力推进体制、机制创新，消除影响经济发展和结构调整的体制性障碍，促进国有资本合理流动与退出，加快国有经济战略性调整。

　　 深化国企改革，调整国有经济布局和结构。抓住国家在我省推行社会保障体系试点的有利时机，全力开展以国有资本和国有职工身份“双退出”为主要标志的国企改革攻坚战，坚定不移地推动国有资本从竞争性领域有序退出。209户竞争性领域市属国有独资中小企业基本完成产权制度改革。9户大型国有独资工商、建工企业和6户公用企业实现投资主体多元化。6户国有控股公司转让国有股权。通过用足用好国家各项扶持政策、出让存量国有资产、盘活土地资源、加大财政支持力度等有效措施，多渠道筹集国企改革所需资金，优先保障职工的合法权益。注重保护现有生产能力，充分安置改制企业职工就业。

　　推进配套改革，减轻企业办社会负担。分步推进企业剥离办社会职能和主辅分离，探索离退休人员统一托管办法，为企业轻装进入市场创造条件。指导企业在实现产权制度改革、理顺职工劳动关系的同时，完善法人治理结构，建立新型劳动用工制度和分配制度，实现制度创新、机制创新和管理创新，增强企业活力。支持集体所有制企业加快改革步伐，实现产权明晰。

　　激活存量，做大总量。充分发挥市场在资源配置中的基础作用，组织企业与国内外大企业、大集团合资合作。以优势产业和骨干企业为依托，重点培育一批以资产为纽带、以产品为龙头、市场竞争力强的大型企业集团。鼓励企业通过境内外直接上市、借壳上市和已上市企业再融资，利用资本市场做大做强。积极调整生产力布局，整合区域资源，整体改造铁北老工业区。

　　 调整完善国有资产管理体制。按照归属清楚、权责明确、保护严格、流转顺畅的现代产权制度要求，建立统一的国有资产管理机构，依法履行出资人职责。探索国有资产监管和经营的有效形式，建立企业经营业绩考核体系，完善授权经营制度。

　　 （二）大力发展非公有制经济，为长春老工业基地振兴注入新的活力。认真清理和修订限制非公有制经济发展的规定和政策，消除体制性障碍，营造有利于非公有制经济发展的良好环境，形成多种经济成分平等竞争、共同发展的新格局。加大扶持措施，使非公有制企业在投融资、税收、土地使用和对外贸易等方面享受与其他企业同等待遇。放宽市场准入条件，引导非公有资本进入法律法规未禁入的基础设施、公用事业及其他行业和领域，拓展非公有制经济的发展空间。改进对非公有制经济的服务和监管，减少行政干预，简化行政管理操作程序，寓管理于服务之中。注重教育引导，依法监督，促进其守法经营、照章纳税，保护职工合法权益。

　　 推进全民创业。在积极争取国家政策和资金支持的同时，坚持自力更生，充分挖掘自身潜力。开展“全民创业、振兴长春”活动，支持群众自主创业，掀起全民创业热潮，共同参与长春老工业基地的振兴。民营企业户数增加20%以上。着力扶持民营经济骨干企业做大规模，突出特色，提高档次，尽快形成一批龙头民营企业。扩大对民营经济的服务，建立完善市、县（区）两级中小企业信用担保体系，发展法律咨询、财务代理等中介服务组织，支持民营企业上市融资、与外资合资合作。引导民营企业加快制度创新、技术创新，鼓励民营企业参与国有企业改组改造。建立公开、透明、畅通的信息网络，使民营企业充分利用资金、技术、人才、信息等各种社会资源，创造社会财富。民营经济增加值达到535亿元，实缴税金28.8亿元，分别增长20%以上。

　　 （三）坚持走新型工业化道路，推进长春老工业基地工业结构优化升级。立足发挥现有的基础、资源和比较优势，坚持有所为、有所不为，主要依靠市场机制作用，促进产业结构调整、生产要素整合及企业改组改造，加快经济结构调整步伐，努力走出一条科技含量高、经济效益好、资源消耗低、环境污染少、人力资源优势得到充分发挥的新型工业化道路。以高新技术产业化和改造提升传统产业为主线，建设大项目，培植大企业，集中力量壮大主导行业，加快传统产业升级。工业增加值达到623亿元，增长15%以上。

　　 举全市之力支持一汽加快发展，做大做强汽车产业。推进19个汽车及零部件重点项目建设，规划建设环一汽100平方公里的汽车产业园区，建设国家级汽车零部件出口基地。一汽轿车新基地形成生产能力，一汽--大众轿车二厂建成焊装、总装、涂装生产线。全市汽车产量达到70.8万辆，增长11%以上。充分发挥长春国家光电子信息产业基地的导向和载体作用，整合光电产业资源，吸引一批高科技企业，启动一批重点项目，尽快形成以光电产业园等6大园区为主体的光电子信息产业基地。启动半导体照明工程，华信光电子光纤通信元器件系统等12个投资超3000万元的光电子信息产业项目竣工投产。光电子信息产业产值达到145亿元，增长20.8%。突出发展现代中药和生物制药，提高自主研发水平。39户制药企业完成GMP改造，产值超亿元的企业发展到16户。生物与医药产业产值达到62.4亿元，增长20%以上。广泛应用高新技术和先进适用技术，加快骨干企业技术改造，重点实施226项投资千万元以上的技改项目。建设和改造投资完成280亿元，增长20.2%。培育主导产品和品牌，提高产品竞争力，扩大市场占有率。500种以上重点新产品投放市场，新产品产值率达到31%以上。

　　 全力抓好项目建设。把项目建设作为老工业基地改造振兴的当务之急和重中之重，摆在政府和企业工作的突出位置，坚持市场选择、企业决策、政府推动相结合，强化组织领导，层层落实责任。按照国家产业政策和投资方向，围绕四大主导行业、现代农业等领域，重点规划设计一批对长远发展有决定性、支柱性和带动性作用的重大项目，努力争取国债支持和社会投资，务求取得突破性进展。全社会固定资产投资完成460亿元，增长18%左右。

　　 （四）统筹城乡协调发展，大力发展现代农业。高度重视“三农”问题，更多地支持农业、关注农村、关心农民，努力缩小城乡差距。围绕富民强县目标，大力推进农村工业化，带动农业集约化、农村城镇化和农民非农化，构筑城乡统筹、工农互动、经济社会生态协调发展的新格局。加速农业结构战略性调整，发展优质、生态、安全的现代农业。突出发展畜牧业，加强资金和政策扶持，推动畜牧业扩大总量和提质提效。推进标准化牧业小区建设，新建标准化牧业小区100个。畜牧业增加值达到90.9亿元，增长12%；畜牧业人均纯收入达到1568元，使之成为农民增收的主要途径。以市场为导向，调整种植业结构，增加优质高产高效粮食、牧草及经济作物种植面积，保护和提高粮食综合生产能力。推进农业标准化进程，完善农产品质量检验检测体系，强化市场准入管理。搞好无公害和绿色食品认证工作，新增无公害、绿色食品基地160万亩。抓好以水利、黑土地保护为重点的农业基础设施建设，发展旱作节水农业，改善农业生产条件。新增节水灌溉面积5万亩。

　　 大力发展县域经济，用工业化思维谋划农业，扩大农业产业化经营。全力培育大成、德大、德莱鹅业等百户龙头企业，构建玉米、肉牛、生猪等10大农产品加工体系，延伸产业链，增加加工深度，尽快壮大第二大支柱产业。启动建设大成德惠生化玉米工业园10万吨赖氨酸、皓月肉牛50万标张皮革加工、华正180万头生猪屠宰等18个投资超3000万元的农产品加工项目。农产品加工业产值达到410亿元，增长17%。发展多种形式的农村专业合作组织，提高农民进入市场的组织化程度。鼓励工商企业打破行业界限，进入农产品加工、流通领域，引进大型营销企业与本地企业合作，形成科研、生产、加工、销售一体化产业链。有步骤地推进城镇体系建设，加快城镇化进程，促进要素集聚，优化农村人口、产业和基础设施布局。

　　 按照“多予、少取、放活”的原则，努力增加农民收入。鼓励农民外出务工经商。取消对农民进城的不合理限制，改善农民进城就业环境，加强法律援助体系建设，保护其合法权益。加强对农村劳动力的培训，增强农民就业和创业能力。农村外出务工经商人数突破80万人次；农民人均纯收入增长8.5%左右。深化农村税费改革，推进对农民补贴方式改革，保护种粮农民利益。依法有序推进土地承包经营权流转，发展适度规模经营。严格执行国家耕地保护的各项政策法规。

　　 （五）坚持改造传统服务业与发展现代服务业并重，积极发展第三产业。把发展服务业作为老工业基地振兴的重要任务，努力提高第三产业在国民经济中的比重。第三产业增加值达到625亿元，增长13%以上。

　　 改造提升传统服务业。对商贸、餐饮等传统服务业，实行优胜劣汰，提高发展水平。集中建设长江路、重庆路、桂林路、红旗街、东盛路等重点商业街区，发展电子商务、物流配送、连锁经营等新型业态和服务方式，促进市场繁荣稳定。全社会消费品零售总额达到485亿元，增长10.7%。建设铁北、东站、汽贸区三大物流园区，培育蔬菜、果品等6个商业配送中心，发展第三方物流企业。

　　 大力发展旅游、会展业。推进重点旅游项目建设，完成伪满皇宫复原工程、净月森林高尔夫球场、长影世纪城和莲花山滑雪场一期建设，启动长春汽车博物馆建设。发展冰雪生态、工农业观光、科技文化和休闲度假等特色旅游，争创“中国最佳绿色旅游城市”。旅游业总收入达到112亿元，增长35%以上。培育办展主体，优化办展环境，精心办好农博会、电影节、民博会、糖酒会、教育展、净月冰雪旅游节暨瓦萨国际滑雪节、国际萨满文化研讨会，打造品牌展会，努力建设北方会展名城。

　　 大力发展新兴服务业和房地产业。对知识密集度高、发挥中介作用的会计、律师、评估、咨询等新兴服务业，边发展边规范，以规范促发展，逐步形成活跃有序的中介服务市场。鼓励中介机构不受地域和所有制限制联合重组，增强服务全省、面向东北的辐射能力。把房地产业作为支柱产业来培育和规范，科学调控经济适用房建设规模，完善供应体制。继续深化住房制度改革，搞活住房二级市场，满足群众住房的需求。房地产投资89亿元，增长14.5%；房地产开发510万平方米，竣工245万平方米。

　　 对涉及国计民生和经济安全的金融、保险、电信等服务业，紧跟国家逐步放宽市场准入的步伐，支持各类社会资本投资入股和参与经营。发挥现有金融机构作用，发展地方商业银行和非银行金融机构。市商业银行完成增资扩股，提高资本充足率。深化农村信用社改革，增强对农村经济支持能力。争取引入国内股份制银行，吸引外资银行来长开展业务。推进银行卡联网通用，做好金融税控收款机试点工作。推进电信普遍服务，促进农村电信消费。电信业增加值达到31.4亿元，增长15%以上。推进财政管理体制改革,健全公共财政体系。依法加强税收征管,强化预算约束机制，严控预算外支出，确保财政收入稳定增长。全口径财政收入达到138亿元，增长2.3％，按可比口径增幅达到13%。

　　 （六）努力扩大就业再就业，进一步加强社会保障体系建设。关心群众切身利益，努力解决困难群众基本生活问题，完善分配制度，进一步提高人民生活水平。城市居民人均可支配收入增长10%左右；城镇登记失业率控制在5%以内。

　　 大力发展劳动密集型产业，充分发挥中小企业和服务业的就业主渠道作用，采用灵活多样的就业方式，不断扩大就业容量，实现发展经济和扩大就业的良性互动。坚持“就业优先”的基本政策取向，尽快形成劳动者自主择业、市场调节就业、政府促进就业的劳动就业机制。全面落实再就业优惠政策，完善就业服务体系，加强对下岗和失业人员职业介绍和再就业培训。全年新增就业岗位7.5万个，安置下岗失业人员5.5万人。对“4050”就业困难群体实施就业援助，安置再就业10000人。实施“扶残助业”工程，安置残疾人就业2000人。加强对高校毕业生、复转军人和城镇新成长劳动力的就业指导和服务。做好劳动用工监察和争议处理工作，切实保障劳动者的合法权益。

　　 把建立健全同经济发展水平相适应的社会保障体系，作为推进改革发展的重要保证措施。认真抓好国家推行社会保障体系试点工作，做实社会养老保险个人帐户，实现国有企业下岗职工基本生活保障向失业保险并轨，确保离退休人员基本养老金按时足额发放。进一步做好城市低保工作，努力做到动态管理、应保尽保。依法扩大养老、失业、医疗、工伤保险征缴覆盖面，重点抓好民营经济等非公有制企业和灵活就业人员的参保工作，织就社会保障的“安全网”，使更多的人老有所养、失有所助、病有所医、伤有所救。推进扶贫帮困工作制度化、社会化、经常化，认真解决困难群众就医、子女就学等问题。

　　 （七）进一步扩大对内对外开放，拓展长春老工业基地发展空间。紧紧抓住国内外生产要素重组、产业转移和中央实施振兴老工业基地战略的机遇，扩大对内对外两个开放，筹集国内国际两类资金，拓展国内国际两个市场，以开放促改革、促调整、促发展。

　　 全力开展招商引资。充分利用中央实施振兴老工业基地战略这面旗帜，积极引进战略投资伙伴，吸引境内外大企业、大集团来长春投资兴业，参与老工业基地的调整改造。突出引资主体。从以政府推动为主尽快转变到以企业为主、政府和社会全面参与上来，激发各类引资主体积极性，形成新一轮招商热潮。突出引资重点。围绕国企改革、四大主导行业和重点大项目特别是国债项目，加大引资力度，确保重点项目顺利实施。扩大引资领域。从以工商业为主拓展到包括公用、社会事业等法律未明令禁止的所有领域，努力营造全方位对外开放的新格局。创新引资方式。从利用外资为主延伸到合作研发、购并重组等国际通行做法上来，提高招商引资效果。实际利用外资9亿美元、内资165亿元人民币，分别增长20%和13.8%。

　　 实施“走出去”战略。鼓励有实力的企业跨国投资和经营，带动产品、劳务和技术装备出口。落实鼓励出口的各项政策，壮大各类出口主体的规模和实力，培育出口生产基地，调整优化出口产品结构，保持外贸出口持续增长。外贸进出口总额达到60亿美元，增长14.9%。加强与东北老工业基地重点城市的联合协作，努力拓展中西部地区和东部沿海地区的市场。

　　 进一步发挥开发区对外开放的龙头带动作用。推进高新、经开、净月、汽贸、长江路5个开发区“二次创业”，创新管理体制和运行机制，优化投资环境，吸引高质量大项目和世界500强企业。引进超千万美元大项目40个以上，国内生产总值平均增长25%以上。全面推进五棵树、合隆等8个开发区建设，编制发展规划，加强基础设施建设，尽快启动一批投资1000万元以上的大项目，形成特色产业的集聚效应。建立健全开发区工作的高位统筹机制，促进全市各开发区各具特色，整体推进。

　　 （八）加强生态环境和基础设施建设，提高城市可持续发展能力。发挥规划龙头作用。完成城市战略规划编制，调整城市总体规划和土地利用总体规划，高标准编制专项规划和控制性、修建性详细规划，做好各类规划申报工作。严格规划审批行政责任追究制度, 强化规划的权威性。

　　 加强城市基础设施建设。实施北人民大街、长春大桥等“九路三桥”工程，整修20条主次干道，改造长农、长吉北线两个城市出入口，建设西安大路等5条标准街路。“轻铁二期”试通车，龙家堡机场试通航，长吉南线等6条公路交付使用。加快公用设施建设与改造步伐，做好后续源水工程的前期准备。认真解决与人民群众关系最密切的热点、难点问题。改造供水管线100公里，综合整治二次供水设施400处。拆迁棚户区房屋35万平方米。严格执行城市拆迁法规，保护被拆迁人的合法权益。实施以租房补贴方式为主的廉租房制度，发放租房补贴700户，廉租房实物配租100户。建成乡村油（水泥）路1000公里以上，改造城区小巷道100条，集中解决130条背街暗巷照明问题。改造燃气管网100公里。推进农村改水改厕，改建城区公厕。

　　 推进“生态城市”建设。合理开发和节约使用各种自然资源，保护生态环境，促进人与自然相和谐。加快恢复波罗湖湿地，推进西部和西北部防风御沙林、“三北”四期防护林建设，启动净月退耕还林工程，植树造林7000公顷。建设伊通河城区中段风光带，改造胜利公园，启动裕华园，续建南湖公园和雕塑公园二期工程。建设自由大路等10条绿化精品街路，完善铁路、三环路沿线绿化带。5城区各新增10块1000平方米以上绿地，建成区新增绿地300公顷。继续改善城市环境空气质量，优良率保持在93%以上。

　　 大力推进污染治理与资源综合利用。全面落实《水污染防治法》，以治理地表水污染为重点，加快城市垃圾、污水处理产业化进程。市属3座污水处理厂达标达效。启动西部“串湖”改造工程，做好北郊“中水回用”和雁鸣湖等区域污水处理工程的前期准备。新建1座粪便无害化处理厂，改造三道垃圾填埋场。抓好医疗垃圾和有毒有害工业废弃物处理。加强机动车尾气和噪声污染治理，建成尾气检测与治理中心。提高使用气体燃料机动车的比例。

　　 创新城市管理体制，完善责权利统一、工作重心下移的城市管理机制。深化土地使用制度改革，规范土地市场管理，依法处理违法用地问题。

　　 （九）实施“科教兴市”战略，为长春老工业基地振兴提供技术支撑和人才保证。强化科技是第一生产力观念，推进科技进步和创新。充分发挥长春高等院校集中、科技力量雄厚优势，实施“振兴老工业基地科技攻关”等政府科技引导计划，推动“产学研”紧密结合，加速科研成果向现实生产力转化。加快高新区吉大科技园、海外学人创业园建设，创办各类科技企业孵化器，建设高新技术创新、中试、孵化基地。坚持引进和自主创新相结合，推动高新技术重大项目的开发、转化和产业化。高新技术产业产值达到650亿元，增长25%以上。产值超亿元的高新技术企业发展到65户。用信息技术改造传统产业，制造业信息化示范企业发展到120户。

　　 围绕办人民满意教育，深化教育体制改革，构建现代国民教育和终身教育体系，努力建设学习型城市。加强基础教育，深化农村办学模式改革，提高农村义务教育的针对性和实用性，下力气解决农村中小学生辍学问题。增加农村教育投入，改造农村倒危校舍8.6万平方米。调整优化高中教育布局，合理确定重点高中、普通高中的招生比例和办学规模，满足不同层次就学需求。发展民族教育和特殊教育。异地新建回族小学，改扩建朝鲜族中学。大力发展职业教育，做大做强职业技术学院，组建职业教育集团。5城区各建1个社区职业教育培训中心。拓展教育社会服务功能，各类学校开展社会培训10万人（次）以上。深化学校内部管理体制改革，推进教师专业化和校长职业化。清理教育乱收费。加强师德建设和德育工作。继续推进高校后勤服务社会化，支持在长高校加快发展。

　　 落实“人才兴国”战略，培养、吸引和用好各类人才，开创人才辈出、人尽其才的新局面。坚持学历教育与继续教育并重，高层次人才与实用型人才培养并重，理论培训与实践锻炼并重，努力培养适应经济社会发展需要的各类优秀人才。在坚持培养本地人才基础上，不断创新引智载体，丰富引智方式，采取多种有效形式，大力引进域外人才。重点抓好急需和紧缺人才、海外留学人员的引进工作。引进各类人才10000人以上。支持知识、智力、技术成果作为生产要素参与收益分配，兑现各项优惠政策, 充分调动各类人才创造财富的积极性。

　　 （十）加强社会主义精神文明建设，推进各项社会事业协调发展。加快推进物质文明、政治文明和精神文明协调发展，促进社会全面进步和人的全面发展。以开展“创建全国文明城市”等活动为载体，以提高市民素质和提升城市文明形象为目标，培育和弘扬城市精神，广泛深入地开展群众性精神文明创建活动，为振兴老工业基地提供积极向上的社会氛围和团结奋进的群众基础。加快新闻、出版、广播、电视以及文化事业改革步伐，创新文化体制和机制。加强文化基础设施建设，鼓励多渠道投入。加快广播电视网络改造。全面完成市图书馆扩建改造工程。繁荣文艺演出市场，丰富群众业余文化生活。加强食品卫生和公共卫生基础设施建设和管理，重点抓好疾病预防控制体系、公共卫生突发事件应急体系和卫生执法监督体系建设。健全防治非典等重大传染性疾病的长效机制。市疾控中心和北方肝胆医院投入使用。积极探索国有医院产权多元化改革，推广新型合作医疗大病统筹试点。贯彻人口与计划生育基本国策，稳定低生育水平，提高人口素质。建立健全市、县、乡、村四级生殖保健服务网络，提高群众的生育质量、生活质量和生命质量。深入开展爱国卫生运动，力争通过“国家卫生城”验收。关心老年人生活，开展好“助老工程”活动。做好妇女儿童、民族宗教、民兵预备役、人防、修志和外事、侨务、对台工作。发展“双拥模范城”成果。

　　 落实《长春2007年亚冬会行动计划》，做好亚冬会各项筹备工作。加快亚冬会比赛场馆、亚运村和新闻中心等设施的规划和建设，改造长春体育中心周边环境。加强群众性体育设施建设，建成 2座体育健身园和30条社区“安居型”健身路径。以“办好亚冬会，建设新长春”为主题，积极承办各类体育赛事。通过举办各种活动，凝聚人心，扩大影响，提高市民的亚冬会意识和参与积极性，为举办亚冬会营造良好的氛围。

　　 大力推进民主政治建设，扩大基层民主，完善公开办事制度，保证人民群众当家作主、依法行使民主权利。整合社区公共资源，完善社区服务功能，建设管理有序、文明祥和的新型社区。推进村民自治，依法组织好第六次村委会换届选举工作。进一步畅通与人民群众密切联系的渠道，认真倾听社会各界对政府工作的意见。建立与工、青、妇及其他群众团体联系制度，积极支持其开展工作。健全市长公开电话网络，加快投诉办理速度，办结率保持在96%以上。

　　 正确处理改革、发展、稳定三者的关系，做到改革力度、发展速度和社会可承受程度有机统一。把人民群众生命安全放在第一位，严厉打击食品、药品、保健品等领域的制假售假行为，抓好农村药品供应、监督、信息网络建设。继续整顿和规范市场经济秩序，严厉查处侵害消费者权益的不法行为。加强对九台营城煤矿沉陷区的综合治理，安排好群众生活。落实安全生产措施，严格执行责任追究制度，坚决杜绝恶性事故发生。坚持“打防结合、预防为主”，落实社会治安综合治理各项措施，完善新型警务运行机制，坚决打击各种犯罪，严厉打击“法轮功”等邪教组织。认真处理群众来信来访，切实为人民群众排忧解难。

　　 （十一）转变政府职能，为长春老工业基地振兴创造良好发展环境。按照“行为规范、运转协调、公正透明、廉洁高效”的总体要求，推进行政管理体制创新，加快政府职能转变。正确处理政府与市场、企业及社会的关系，把属于企业的生产经营权和投资决策权交给企业，把社会可以自我调节和管理的职能交给社会中介组织，把群众自治范围内的事情交给群众，把政府经济管理职能转到主要为各类市场主体服务和创造良好发展环境上来。

　　 坚持依法行政。深入贯彻《行政许可法》，全面清理不符合规定的行政许可，该废止的坚决废止，该修订的抓紧修订，该强化管理的强化管理，力争上半年完成清理任务。加强对行政执法的监督，着手建立公民、法人和其他组织对违法和不当行政决定的申诉、检举制度。清理行政事业性收费。取消没有上位法依据而自行设立的收费项目，降低名义收费高于实际征收的收费标准，剥离不属于行政事业性范围的收费项目，规范细化弹性收费标准，取消或缩小收费自由裁量权，严肃处理违法违纪收费行为。 推行收费公示制度，向社会公开《行政事业性收费管理目录》，从源头上取缔乱收费行为。加强 “收支两条线”管理，实现收缴分离和收支脱钩。整体收费标准力争达到东北副省级城市最低。

　　 建设服务型政府。把提高服务质量和企业、公众对政府所提供服务的满意度，作为衡量政府行政效率和水平的主要标准。不断修订、补充、完善服务项目和措施，最大限度地方便市民、方便企业、方便社会。完善政务中心服务功能，实行首办窗口负责制，扩大“一站式”服务范围，减少审批环节，提高办事效率。加快政务中心电子政务建设，实现审批微机管理、全程监控。政务中心审批事项当天办结率再提高4个百分点，达到90%以上。完善政府信息公开和新闻发言人制度，定期举行新闻发布会和实行网上公开，及时发布政务、政策信息，为社会提供信息服务，保证公民的知情权。

　　 转变政府工作作风。全面精减会议和文件，少开会、开短会，少发文、发短文，力戒形式主义。全面清理和取缔劳民伤财、没有实际意义的各种检查、评比、达标活动，实行严格的检查报批和社会公布制度，增强检查的透明度。严控各类庆典和表彰活动。坚持深入基层，加强调查研究，解决实际问题，推动工作落实。

　　 推进政府廉政建设。认真落实党风廉政建设领导干部责任制和廉洁自律规定，深入开展纠风专项治理，严肃查办大要案。强化制度约束，从源头上预防和治理腐败。完善政府采购制度，扩大市、县（区）联合采购规模，把使用财政性资金的工程建设、物资采购、中介机构选用，全部纳入政府采购范围。加强政府采购信息化建设，实现全流程网上采购。推进部门预算管理制度改革，把市直预算外事业单位纳入部门预算管理。扩大会计集中核算范围，把32家市直部门纳入第二批会计集中核算体系。严肃财经纪律，清理整治“小金库”。全面实行建设工程招投标，完善土地收购储备、招标拍卖、挂牌出让制度，杜绝“暗箱操作”。

　　 提高政府工作科学化、民主化、法制化水平。凡属经济社会发展的重大决策，均通过市政府决策咨询机构组织有关专家充分论证。凡涉及群众切身利益的重大决策，均举行听证会。健全监督机制，全面推行行政执法责任制，严格落实责任追究制。发挥监察和审计机关作用，组织暗访或音像实录，开展“百名处长社会公开评议”、“人民满意公务员和集体评选”等活动，表彰先进，帮促后进。切实发挥软环境投诉中心作用，做到有诉必查、有错必纠、查实必处。自觉接受人大的法律监督、工作监督和政协的民主监督，依法向人大、政协报告和通报工作。建立市政府组成人员与人大代表、政协委员联系制度。认真办理人大代表建议和政协委员提案，不断提高办理质量。虚心接受新闻舆论的监督。

　　 各位代表！我们已经踏上了振兴老工业基地、全面建设小康社会的伟大征程。让我们紧密团结在以胡锦涛同志为总书记的党中央周围，在省委、省政府和市委的领导下，解放思想，实事求是，与时俱进，开拓创新，为实现经济社会全面、协调、可持续发展，创造长春的美好未来而努力奋斗！
